# Physical exercise and intermittent administration of lactulose may improve autism symptoms through hydrogen production

**DOI:** 10.1186/2045-9912-2-19

**Published:** 2012-07-30

**Authors:** Ahmad Ghanizadeh

**Affiliations:** 1 Research Center for Psychiatry and Behavioral Sciences, Department of Psychiatry, School of Medicine, Shiraz University of Medical Sciences, Shiraz, Iran; 2 Department of Psychiatry, Shiraz University of Medical Sciences, School of Medicine, Shiraz, Iran

**Keywords:** Lactulose, Hydrogen, Autism, Oxidative stress, Exercise, Therapy

## Abstract

Autism is neuro-developmental disorder. Oxidative stress is enhanced in some children with autism. Hydrogen is a gas with anti-oxidative effects suggested for treating or prevention of some medical problems. It is hypothesized that lactulose or hydrogen water may provide hydrogen to reduce oxidative stress in autism.

## Introduction

### Autism and oxidative stress

Autism is a neurodevelopmental disorder that its diagnosis is increased in recent decade. In addition to increasing professionals and public awareness regarding autism, its diagnostic concept is broaden [1]. According to parents’ report and DSM-IV diagnostic criteria, about 1.9% of a sample of school aged children in Iran meet the screening cutoff scores for possible autistic disorder [2]. The neurobiology and genetics of autism is not clearly known [3].

Oxidative stress probably plays a significant role in the neurobiology of autism [4]. Some symptoms of autism are mediated by oxidative stress [5]. Urinary oxidative stress markers levels in autism are more than that of the controls and these levels are associated with the severity of autism [6]. Lipid peroxidation is increased in autism [7]. The vulnerability of children with autism to oxidative stress is higher than that of the controls [8]. For example, the total and reduced levels of glutathione are decreased in some children with autism, while reactive oxygen species and oxidized glutathione level are increased [9]. Moreover, the activity levels of glutathione- s-transferase and catalase are decreased [9]. Besides, the serum levels of transferrin and ceruloplasmin as significant antioxidant proteins are decreased in autism [7].

Brain-derived neurotrophic factor (BDNF) in brain in some children with autism is reduced [10]. Sonic hedgehog (SHH) protein and brain-derived neurotrophic factor (BDNF) are associated with oxidative stress in autism [11]. This association might be mediated by Malondialdehyde, Bcl-2, superoxide dismutase and glutathione peroxidase [12].

The increased oxidative stress is suggested as a treatment target to improve the function of mitochondria [13-15]. Many of the suggested treatments targeted neuro-inflammation [16,17]. Already, some uncontrolled studies reported that hyperbaric oxygen is beneficial for some children with autism. However, there are not enough evidence for its efficacy for treating autism [18]. There is a limited number of FDA approved medications for treating autism. Finally, while there is no curative treatment for autism, alternative medications are highly required.

### Hydrogen and oxidative stress

Hydrogen molecule defenses against oxidative stress [19]. There are two advantages for hydrogen molecule. First, it decreases the hydroxyl radical which is a potent cytotoxic reactive oxygen species. Secondly, Hydrogen does not react with other reactive oxygen species. This is very important because some of the reactive oxygen species have physiological roles [20]. The therapeutic effects of hydrogen is reported for some problems, such as hepatic injury [21], atherosclerosis prevention [22], and isplatin-induced ototoxicity [23]. Recently, it has been suggested that hydrogen may decrease some symptoms of autism [24].

### Lactulose and hydrogen

Lactulose is not absorbed from intestine in humans but this synthetic sugar can be used by some intestinal bacteria to produce hydrogen. Hydrogen in breath air is increased after taking lactulose [25]. However, intermittent hydrogen administration more than continually increased level of hydrogen is effective for protection against Parkinson disease [25]. The effect of hydrogen water is more effective than administering lactulose or continually administration of 2% hydrogen gas [25]. In fact, the effect of intermittent administration of hydrogen is more than continually administration of hydrogen. In other words, the dose of hydrogen is not important as much as being administered intermittently [25]. Bacteria in human gut can produce hydrogen from lactulose that exercise exacerbates this hydrogen production [26].

In addition, about 42% of children with autism suffer from gastrointestinal problems [27]. Both constipation and diarrhea are the most common problems [27]. The incidence rate of constipation in autism is 33.9% while this rate for the control group is 17.6% [28]. Not only constipation is a common gastrointestinal problem in children with autism but also constipation is associated with increased social impairment and lack of expressive language [29]. Therefore, there is a need for treating constipation in autism [29]. It is supposed that there is a common genetic factor for intestinal and behavioral problems in autism that targeting both problems is recommended for treating autism [30]. Lactulose is a osmotic laxative which is used for treating constipation [31].

### Physical exercise and autism

While the physical activity of children with autism is lesser than the controls [32], physical exercise decreases some of the symptoms of autism. Exergaming which is a simultaneous combine of physical and mental exercise decreases stereotypies in autism [33]. Moreover, exergaming enhances their cognitive function [33]. In addition, aerobic exercise improves academic function of children with autism [34]. Besides, the improved motor proficiency and sensory integrative functions after physical activity are sustained for long term [35].

### Hypothesis

There are many reports about the role of oxidative stress in autism. Moreover, hydrogen is a gas with anti-oxidative stress effects. There are some resources for hydrogen, such as hydrogen water and lactulose. Lactulose administration enhances hydrogen production in intestines. Considering that oxidative stress is a target for treating autism, it is hypothesized that lactulose may target both oxidative stress in brain and constipation in autism. Therefore, it is worthy to investigate the possible effect of lactulose or hydrogen water on autism. Taking into account that continues hydrogen is less effective than intermittent hydrogen administration, intermittent lactulose administering should be considered.
